# Common cues to emotion in the dynamic facial expressions of speech and song

**DOI:** 10.1080/17470218.2014.971034

**Published:** 2014-11-25

**Authors:** Steven R. Livingstone, William F. Thompson, Marcelo M. Wanderley, Caroline Palmer

**Affiliations:** ^a^ Department of Psychology, McGill University, Montreal, QC, CanadaH3A 1B1; ^b^ Department of Psychology, Macquarie University, Sydney, NSW, Australia; ^c^ Department of Music Research, CIRMMT, McGill University,Montreal, QC, Canada

**Keywords:** Facial expressions, Emotion, Dynamic information, Vocal communication, Speech, Singing

## Abstract

Speech and song are universal forms of vocalization that may share aspects of emotional expression. Research has focused on parallels in acoustic features, overlooking facial cues to emotion. In three experiments, we compared moving facial expressions in speech and song. In Experiment 1, vocalists spoke and sang statements each with five emotions. Vocalists exhibited emotion-dependent movements of the eyebrows and lip corners that transcended speech–song differences. Vocalists’ jaw movements were coupled to their acoustic intensity, exhibiting differences across emotion and speech–song. Vocalists’ emotional movements extended beyond vocal sound to include large sustained expressions, suggesting a communicative function. In Experiment 2, viewers judged silent videos of vocalists’ facial expressions prior to, during, and following vocalization. Emotional intentions were identified accurately for movements during and after vocalization, suggesting that these movements support the acoustic message. Experiment 3 compared emotional identification in voice-only, face-only, and face-and-voice recordings. Emotion judgements for voice-only singing were poorly identified, yet were accurate for all other conditions, confirming that facial expressions conveyed emotion more accurately than the voice in song, yet were equivalent in speech. Collectively, these findings highlight broad commonalities in the facial cues to emotion in speech and song, yet highlight differences in perception and acoustic-motor production.

Throughout history, speech and song have served as overlapping and interchangeable forms of vocal expression. In the Western classical tradition, *Sprechstimme* refers to a stylized form of vocal expression halfway between singing and speaking (Owen, Ellen, David, & John, 2012), while in ancient Greece, the words *singing* and *speaking* were used interchangeably (Stamou, 2002). A significant body of research has focused on acoustic cues, identifying overlaps in the expression of emotion between speech and song (Cowie et al., 2001; Gabrielsson & Lindström, 2001; Ilie & Thompson, 2006; Juslin, 2001; Juslin & Laukka, 2003; Scherer, 1995, 2003). This emphasis on the acoustic modality, however, overlooks the role of dynamic facial cues to emotion in speech and song (Davidson, 1993).

Visual gestures of great performers, including facial expressions and body movements, complement the voice signal, communicating through motion. Performers’ facial expressions are likely to play an important role in vocal communication as emotion is often identified more accurately from visual gestures than from acoustic signals (Davidson, 1993; Elfenbein & Ambady, 2002). However, relatively little is known about the role of facial expressions in vocal performance (Livingstone, Thompson, & Russo, 2009; Thompson, Russo, & Quinto, 2008).

Vocalization places demands on orofacial motion (Craig, van Lieshout, & Wong, 2008; Lindblom & Sundberg, 1971; Sundberg & Skoog, 1997) that distinguish vocal facial expressions from their prototypical silent counterparts. Motor actions for vocalization complicate the study of movements tied to emotional expression. For example, rounding of the lips is required for the production of the phoneme /w/ such as in who'd (/hu:d/) or going (/ɡoʊɪŋ/; Fernald, 1989), and pursing of the lips is needed for the production of /b/ as in bank (/bæŋk/). Simultaneously, facial expressions of happiness are often expressed with a raising, broadening, and pulling back the lip corners (Darwin, 1872/1965, p. 199; Kohler et al., 2004). To control for phoneme-specific interactions with emotion, we examined vocalizations of full statements rather than individual vowels (Carlo & Guaitella, 2004).

Facial expressions of emotion during vocalization are expected to be similar to their nonvocal emotional counterparts: Happiness should be expressed with a raising of the lip corners and eyebrows, and sadness should be expressed with a furrowing of the eyebrows and a slight depression of the lip corners (Kohler et al., 2004). It is unknown how vocalized emotion will affect vocalists’ jaw movement. Motion of the jaw is tightly coupled to sound production, where a wider jaw opening has been associated with increased vocal intensity and a faster speech rate (McClean & Tasko, 2003; Tasko & McClean, 2004). These two qualities are also associated with emotional expression, in which a higher vocal intensity and faster rate/tempo are typically associated with happiness, and a lower intensity and slower rate are associated with sadness (Cowie et al., 2001; Kotlyar & Morozov, 1976; Scherer, 2003; Sundberg, Iwarsson, & Hagegård, 1995). We hypothesized that jaw motion would differentiate emotional expressions during speech and song, with happy expressions exhibiting a wider jaw opening than sad expressions. Differences in the acoustic features of intensity and rate have also been reported as varying between speech and song, where singing exhibits a louder vocal intensity, but a slower rate (Livingstone, Peck, & Russo, 2013). We explored these hypotheses in Experiment 1 with an examination of lip corner, eyebrow, and jaw motion during happy and sad emotional productions of speech and song.

An important aspect of how vocal facial expressions convey emotion may lie in the timeline of expressive movement. The presence of dynamic information in facial expressions has been shown to improve observers’ accuracy of emotion recognition, judgements of emotional genuineness, and the accuracy of speaker identity (Ambadar, Cohn, & Reed, 2009; Atkinson, Dittrich, Gemmell, & Young, 2004; Bassili, 1978, 1979; Bugental, 1986; Cunningham & Wallraven, 2009; Kamachi et al., 2001; Krumhuber & Kappas, 2005; O'Toole, Roark, & Abdi, 2002). Livingstone et al. (2009) found that singers’ expressive facial movements lingered for up to 3 seconds after the end of vocalization. These movements may convey significant emotional information and may therefore be a general property of communication in both speech and song. We hypothesized that emotion-dependent extravocal movements would be present in both speech and song and would convey significant emotional information to observers. We explored these hypotheses in Experiment 2, by examining observers’ perception of emotion from vocalists’ facial expressions occurring prior to, during, and following vocal sound.

Facial expressions are likely to play an important role in vocal communication due to their accuracy in conveying emotion. In a review of Western and cross-cultural studies, Scherer (2003) concluded that facial expressions of emotions are identified on average with 75% accuracy, while verbal and nonverbal acoustic expressions are identified with 55% to 65% accuracy (see also Elfenbein & Ambady, 2002). Studies of music performance have reported similar findings, where the visual performance often conveys emotion more accurately than the sounded performance (Carlo & Guaitella, 2004; Davidson, 1993; Vines, Krumhansl, Wanderley, Dalca, & Levitin, 2011). However, little is known about the effectiveness of facial expressions during vocal sound production. Which is more accurate at conveying emotion during vocal communication, the face or the voice? We hypothesized that facial expressions of emotion would be identified more accurately than vocal expressions in speech and in song. We explored this hypothesis in our third experiment, with a comparison of observers’ perception of emotion from speech and song. We also questioned whether the combination of audio information with visual facial expressions would affect emotion recognition rates. Previous studies have reported mixed results, in which the addition of vocal content sometimes improved recognition rates over visual-only content (Elfenbein & Ambady, 2002). Therefore, we expected that recognition rates for full audiovisual presentations in Experiment 3 should be at least as high as those for visual-alone presentations and higher than those for audio-alone presentations.

Three experiments examined the dynamic nature of facial expressions in speech and song. The first experiment examined the facial movements of vocalists who spoke and sung short phrases with different emotions. We expected that facial expressions would show characteristic emotion-related patterns that transcended lexical variability in movements of lips and eyebrows and showed movements of the jaw that differentiated emotional expression. The second experiment examined viewers’ perception of emotion during the timeline of expressive vocalization. Observers identified emotion of vocalists from silent videos showing movements prior to vocal onset, during vocalization, and after vocalization had ended. We expected that emotions would be identified accurately for facial movements during and after vocalizations. The third experiment compared the influence of visual (facial), auditory (vocal), and auditory–visual cues on observers’ perception of emotion during vocalization. We expected that audio-only presentations would be identified least accurately, in both speech and song.

## EXPERIMENT 1

Participants were required to speak or sing short statements with different emotional intentions (very happy, happy, neutral, sad, and very sad) while their facial motion and vocal productions were recorded. We predicted that facial motion of vocalists would change with emotional intentions, above and beyond lexical stimulus differences. We expected that happiness would be expressed with raised lip corners and raised eyebrows; and that sadness would be expressed with furrowed eyebrows (Kohler et al., 2004). We further expected that happiness would exhibit a greater opening of the jaw than sadness, due to differences in vocal intensity (McClean & Tasko, 2003).

### Method

#### Participants

Twelve adult participants (mean age = 23.4 years, *SD* = 5.7, 6 females) were recruited from the Montreal community. Participants were native English speakers and had at least six years of vocal experience (*M* = 9.83 years, *SD* = 3.0) and varied amounts of private vocal instruction (*M* = 6.83 years, *SD* = 4.8). Participants were screened to ensure they had not received prior training on how to move or hold the face while singing.^1^ The experiment took approximately 90 minutes, and participants received a nominal fee for their participation.

#### Stimulus

Four neutral English statements were used (“People going to the bank”, “Children tapping to the beat”, “Children jumping for the ball”, and “People talking by the door”). Statements were seven syllables in length and were matched in word frequency and familiarity using the MRC (Medical Research Council) psycholinguistic database (Coltheart, 1981). In the song condition, an isochronous melody (F4, F4, G4, G4, E4, E4, F4; piano MIDI tones) consisting of six-eighth notes (300 ms) and ending with a quarter note (600 ms), was used. The melody did not contain the third scale degree and was designed to be ambiguous in terms of a major or minor mode, which are often associated with happy and sad emotions, respectively (Dalla Bella, Peretz, Rousseau, & Gosselin, 2001; Hevner, 1935).

#### Apparatus

Stimuli were presented visually on a 15″ Macbook Pro and auditorily over Sennheiser HD 500 headphones, controlled by Matlab and the Psychophysics Toolbox (Brainard, 1997). Temporal accuracy of the presentation software was confirmed with the Black Box Toolkit. An active motion capture system (NDI Optotrak Certus; spatial accuracy 0.1 mm) monitored the facial movements of participants at a frame rate of 250 Hz. Three-mm markers were placed symmetrically on the left and right lip corners (zygomaticus major), inner and middle eyebrows (corrugator supercilii), under the eyes (orbicularis oculi), above the lips (philtrum), and below the lips between the orbicularis oris and mentalis. Additional markers on each participant's headphones (headband and left and right earcups) provided a rigid body with which to align the motion coordinate system. Vocal utterances were captured with an AKG C414 B-XLS cardioid microphone, placed 1.5 m in front of the vocalists, at 44 kHz. Sound recordings were synchronized with motion data via the Optotrak Data Acquisition Unit.

#### Design and procedure

The experimental design was a Channel (2 levels: speech, song) × Emotion (5 levels: neutral, happy, very happy, sad, very sad) × Statement (4) × Repetition (2) within-subjects design, with 80 trials per participant. Trials were blocked by channel, with speech presented first to avoid any temporal influences from the regular pace of the song condition. Trials were blocked by emotion category (happy, neutral, or sad) and statement, with normal emotions followed by their very intense counterparts (e.g., happy then very happy). Trials were blocked by emotion to allow vocalists to enter into and remain within the desired state for all productions of the emotion.

**Figure 1:**
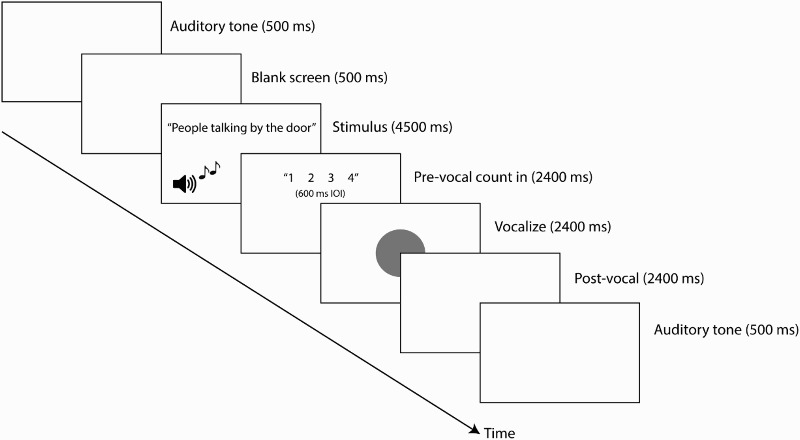
Timeline of trials in Experiment 1. Each trial began with a 500 ms auditory tone, followed by 500 ms of blank screen. The statement to be spoken or sung was then presented. In the song condition, the melody was also sounded. A pre-vocal count-in timer was then presented. Participants began vocalization with the appearance of the green circle. Additional movements were captured during the post-vocal epoch (blank screen). The trial ended with a 500 ms auditory tone. Facial motion and acoustic information was captured throughout the entire trial timeline.

Participants were told to prepare themselves emotionally as they would for a live performance and were given time between blocks to prepare themselves. Vocalists were given no instruction regarding their facial composure leading up to or following the offset of vocal sound and were told only to speak or sing in “an expressive manner as though performing to an audience”. Participants began with a series of speech practice trials; the statements used differed from those presented in the experimental trials. The trial timeline, presented in Figure 1, consisted of four main epochs: stimulus presentation (visually displayed statement), count-down timer (4–3–2–1), begin vocalization (green light), and end of vocalization. Practice trials were repeated until participants were comfortable with the task. Participants were first shown the four statements that would be used throughout the experiment. Participants then completed the speech experimental block. At the end of the speech trials, after a rest break, participants completed a series of song practice trials (with the same statements as those in the speech practice trials). In the song condition, participants were told to sing one syllable per tone, using the pitches and timing of the presented melody—for example, peo(1)-ple(2) talk(3)-ing(4) by(5) the(6) door(7). Trials were repeated if participants made a mistake, or if they moved outside the motion capture volume.

#### Analyses

Head motion data were transformed (rotation + translation) to a local coordinate system of the participant's head using a three-marker rigid body formed by the principal axes of the participant's headphones. Reference markers on the participant's headphones provided a rigid body that enabled us to define a new local coordinate system. This transformation enabled the analysis of individual facial features in the six degrees of freedom of motion (6DoF). An analysis that considers six degrees of freedom is critical to the study of facial expressions, where it is the direction of facial feature motion that differentiates emotions (e.g., raised versus furrowed eyebrows, a smile versus a frown). The approach represented an important methodological improvement over “point-to-point” Euclidean distance analysis (1DoF) which reflect the magnitude but not the direction of movement.

Marker positions were individually set to baseline values of a “neutral resting” position of the participant's face. Marker data were zeroed using a baseline subtraction procedure. A baseline window of 2000 ms prior to each trial onset was selected. For each marker, the modal value within the baseline window was subtracted from marker displacement during the trial timeline. These baseline-adjusted marker trajectories represented how the marker deviated throughout the trial from its resting position.

We analysed vertical motion of the lip corners, as this is the dimension of motion typically described in the facial expression literature. We analysed vertical and horizontal displacement for the left eyebrow, as both dimensions are commonly described in the facial expression literature. We analysed the Euclidean displacement of the jaw. The jaw rotates around the terminal hinge axis, with motion occurring primarily in the sagittal plane defined by the vertical (up–down) and depth (back–forward) axes, with limited horizontal (side-to-side) motion (Edwards & Harris, 1990). Thus, Euclidean distance simplifies the analysis of jaw motion by reducing it to a single dependent variable, while capturing the full range of motion.

Motion data were analysed with functional data analysis techniques (Ramsay & Silverman, 2005), which model discrete data as a continuous function. Feature registration across trials was used to enable the statistical comparison of unequal duration trajectories by aligning data using temporal event landmarks at the boundaries of the four timeline epochs. Occasional missing data were interpolated (less than 0.0001% of data), and order 6 B-splines were fitted to the second derivative of marker trajectories with a ratio of 1:4 knots to data samples. The data were smoothed using a roughness penalty on the fourth derivative (*λ* = 10^−8^). Feature registration was also used to temporally align trajectories to the syllable boundaries (6 events) as determined from the acoustic analyses, described below. To enable comparisons of syllable trajectories across utterances, the functional data were resampled to produce 75 equally spaced data points per syllable (300 ms at 250 Hz) for the first six syllables, with the final syllable resampled to 150 data points (600 ms at 250 Hz). Thus, data were resampled from the continuous function within each epoch to generate equivalent numbers of data points for each trial; this derivation enabled a syllable-matched comparison across speech and song. Functional analyses of variance (fANOVAs) were used to examine motion trajectories at each time point across the entire trial (see also Livingstone, Palmer, & Schubert, 2012). Functional ANOVA tests for statistical differences at every resampled time point in the functional data. Significance levels were corrected for multiple comparisons with false discovery rate using the Benjamini–Hochberg–Yekutieli procedure for dependent statistical tests, with a *q*-value of 0.05 (Benjamini & Hochberg, 1995; Benjamini & Yekutieli, 2001). We report mean *F*-statistic and mean eta-squared values across time regions that reached statistical significance (*p* < .05). Effect sizes are reported as eta-squared values.

Acoustic recordings were analysed with Praat (Boersma & Weenink, 2010). Utterances were segmented at syllable boundaries and were coded by a rater; 8% of the samples were checked by a second rater (mean interrater boundary time difference = 0.0026 s, *SD* = 0.0024 s). Syllable boundaries were determined by changes in the spectrogram and in the fundamental frequency and acoustic intensity contours. Eight syllable boundaries were determined in each utterance, and time values of these boundaries were used as event landmarks in the functional data registration. The parameter values and functional landmarks used in analysis of the motion data were reused for the acoustic intensity contour data, with a roughness penalty of *λ* = 10^−14^.

### Results

#### Lip corner data

**Figure 2:**
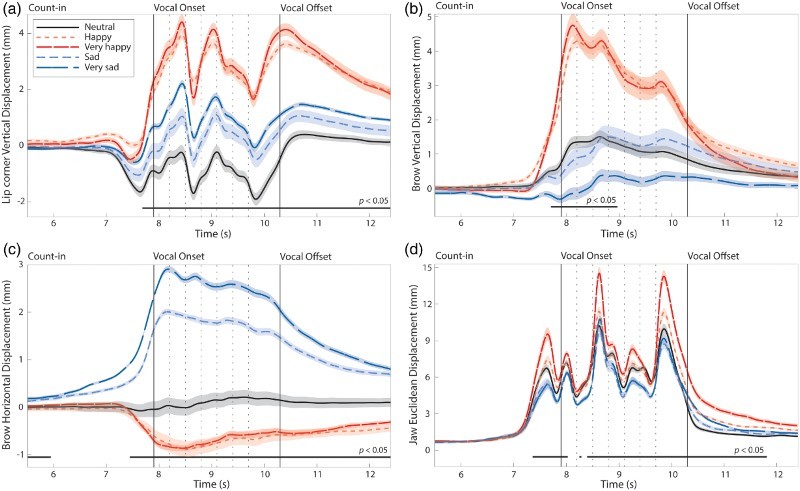
Main effects of Emotion conditions on four aspects of facial motion in Experiment 1. Each trajectory line is the functional, time-aligned mean across all actors, vocal channels, statements, and repetitions (192 trials per trajectory line). Zero represents the neutral “at rest” position of the facial feature. Dashed vertical lines between Vocal Onset and Offset indicate syllable boundaries. Black horizontal lines below trajectories indicate regions of significance at *p* < 0.05. Error bars are indicated by shaded regions around trajectory lines, where error bars denote the standard error of the means. (a) Mean vertical displacement of left lip corner. (b) Mean vertical displacement of left brow. (c) Mean horizontal displacement of left brow. (d) Mean Euclidean displacement of the jaw.

A three-way fANOVA on the vertical lip corner displacement measures (deviation from resting position of face) by channel (2 levels: speech, song), emotion (5 levels: very happy, happy, neutral, sad, very sad), and statement (4) was conducted. No effect of channel or its interactions were found in the analysis of vertical lip corner motion. Figure 2a shows the mean lip corner displacement values across all trials by emotion conditions. Regions of statistical significance (*p* < .05) are indicated by the black horizontal bar in the timeline. The main effect of emotion lasted from before the onset of vocal production and continued throughout the vocalize and postvocal epochs, 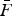
(4, 44) = 13.25, *p* < .05, 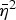 = .26. Happy emotions were characterized by larger vertical motion of the lip corners than that for sad emotions. Neutral utterances appeared to exhibit the smallest level of vertical lip corner movement. A main effect of statement was indicated during the majority of vocal sound (1684 ms of 2400 ms = 70.2%), as would be expected due to the pronunciation of varying phonemic content, 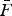
(3, 33) = 14.57, *p* < .05, 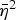 = .07. A significant Statement × Emotion interaction was also reported for 300 ms of the epoch containing vocal sound, 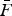
(12, 132) = 3.72, *p* < .05, 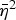 = .02, indicating that the effect of lexical content on vertical lip corner motion was mediated by emotion. These results confirm that lip corners differed across emotions, but did not differ across speech and song. As expected, expressive lip corner movements continued after the end of vocalization in both speech and song.

#### Eyebrow data

Separate three-way functional ANOVAs on the vertical and horizontal eyebrow displacement measures by channel (2), emotion (5), and statement (4) were conducted. No effect of channel or its interactions were found in the analyses for either vertical or horizontal brow motion, confirming that speech–song differences did not affect vocalists’ brow motion. Figures 2b and 2c show the mean values for vertical and horizontal brow displacement, respectively, by emotion condition. A significant main effect of emotion was reported for vertical brow motion, 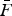
(4, 44) = 10.26, *p* < .05, 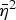 = .23. Happy emotions elicited large vertical rising of the eyebrows, beginning 240 ms prior to vocal onset and continuing for 1152 ms of the epoch containing vocal sound (48%). These results confirm that vertical brow motion differentiated the vocalists’ emotions and occurred in the earlier stage of vocalization. No effects of statement or its interactions were recorded for vertical brow motion.

A significant main effect of emotion was reported for horizontal brow motion, 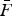
(4, 44) = 22.77, *p* < .05, 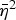 = .38. Sad emotions elicited large inward horizontal motion, producing characteristic furrowing of the eyebrows. Significant brow motion began immediately after stimulus presentation (count-in) and continued throughout the entire vocalization and postvocal epochs. No effects of statement or its interactions were recorded for horizontal brow motion. These results suggest that emotion-dependent eyebrow movements transcended speech–song and lexical differences.

#### Jaw data

**Figure 3:**
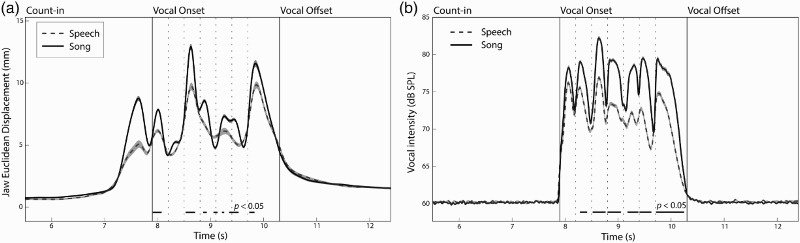
Main effect of Channel (speech/song) condition on (a) Mean Euclidean displacement of the jaw across all actors, emotions, statements, and repetitions (480 trials per trajectory line), and (b) Mean acoustic intensity across all actors, emotions, statements, and repetitions. Dashed vertical lines between Vocal Onset and Offset indicate syllable boundaries. Black horizontal lines below trajectories indicate regions of significance at *p* < 0.05. Error bars are indicated by shaded regions around trajectory lines, where error bars denote the standard error of the means. Note: *The large peak in jaw motion prior to the onset of vocal sound reflects the inhalation of breath by vocalists*.

A three-way functional ANOVA on Euclidean jaw displacement measures by channel (2), emotion (5), and statement (4) was conducted. Figure 3a shows the mean Euclidean jaw displacement values across all trials by channel conditions. A main effect of channel was found for 828 ms of the vocalize epoch (34.5% of 2400 ms), with song exhibiting a wider jaw opening than speech, 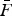
(1, 11) = 16.06, *p* < .05, 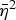 = .07. Figure 2d shows the mean Euclidean jaw displacement for the emotion conditions. A main effect of emotion began shortly before the onset of vocal production, occurred for 1992 ms of the vocalize epoch (2400 ms, resampled time), and continued for 1300 ms of the postvocal epoch (2100 ms, resampled time), 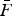
(4, 44) = 12.47, *p* < .05, 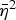 = .09. As hypothesized, happy vocalizations appeared to exhibit a wider opening of the jaw than sad vocalizations. A large motion peak was also recorded prior to the onset of vocalization, as shown in Figure 2d. Inspection of the video camera and sound recordings confirmed that this motion peak reflected inhalation by the participants before the start of vocalization.

A main effect of statement was found through 1960 ms of the vocalization epoch, reflecting expected differences in lexical articulation, 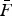
(3, 33) = 27.94, *p* < .05, 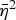 = .23. A Channel × Statement interaction occurred for 888 ms of the Vocalize epoch, 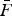
(3, 33) = 16.42, *p* < .05, 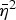 = .05, and a Channel × Emotion interaction occurred for 100 ms of the vocalize epoch, respectively, 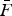
(4, 44) = 8.06, *p* < .05, 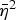 = .02. A Statement × Emotion interaction occurred for 816 ms of the vocalize epoch, 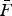
(12, 132) = 3.49, *p* < .05, 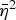 = .02, which occurred primarily in the last syllable of the utterance. These results indicate that the effect of channel was mediated by both the statement and, for a briefer period, the emotion of the utterance. Overall, these results confirm that motion of the jaw is tightly coupled to sound production, reflecting differences in both acoustics and phonemic content.

We next examined acoustic intensity to determine whether loudness of the voice may explain observed differences in jaw motion between speech and song, and across the emotional conditions. A two-way fANOVA on acoustic intensity by channel (2) and emotion (5) was conducted. Figure 3b shows the acoustic intensity values across all trials by channel conditions. A main effect of channel was found for 1592 ms of the vocalization epoch (66% of 2400 ms), 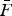
(1, 11) = 30.81, *p* < .05, 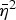 = .25. Differences in jaw motion for speech and song fell within these time regions. A main effect of emotion was found for 2370 ms of the vocalization epoch (99% of 2400 ms), 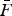
(4, 44) = 11.28, *p* < .05, 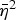 = .16. A Channel × Emotion interaction was also found for 360 ms, 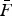
(4, 44) = 7.27, *p* < .05, 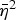 = .02. Differences in Channel × Emotion jaw motion primarily fell within these time regions, as shown in Figure 3. A correlation between mean Euclidean jaw displacement and mean acoustic intensity measures during the vocalize epoch was highly significant, *r*(599) = .408, *p* < .001.^2^ These results suggest that differences in jaw motion between the emotional conditions, and speech and song, were due in part to differences in the acoustic intensity across emotions.

### Discussion

Vocalists exhibited emotion-dependent facial movements that overcame lexical variability and speech–song differences. Happy expressions were characterized by raised lip corners and raised eyebrows and a wider opening of the jaw. Sad expressions were characterized by inward furrowing of the eyebrows and a smaller opening of the jaw. Neutral emotions were conveyed through a general attenuation of movement and a slight depression of the lip corners. Movements of the lip corners and eyebrows match those reported in the literature for prototypical, nonvocal expressions of happiness and sadness (Kohler et al., 2004). As hypothesized, vocalists’ facial expressions also differed in motion of the jaw across emotional conditions.

Vocalists’ jaw motion exhibited emotion-dependent and channel-dependent differences throughout vocalization. An analysis of the acoustic signal revealed that vocalists’ jaw motion was positively correlated with their vocal intensity (McClean & Tasko, 2003; Tasko & McClean, 2004). Happy vocalizations exhibited a louder vocal intensity and wider jaw opening, while sad vocalizations exhibited a lower intensity and smaller opening of the jaw. Similarly, song exhibited a louder vocal intensity and wider opening of the jaw relative to speech. These findings suggest that differences in motion of the jaw across the emotional and channel conditions partly reflect differences in the acoustic intensity between these conditions. These results identify the jaw as a new facial feature in the expression of happy and sad vocal emotions (Ekman & Friesen, 1978). As expected, motion of the jaw exhibited strong differences across the four statements, reflecting the tight coupling between jaw motion and acoustic production. A large opening of the jaw was also reported prior to the onset of vocal sound, reflecting inhalation prior to sound production. Differences in jaw opening across emotions may reflect a greater inhalation of air for louder emotions, as air flow rate is correlated with vocal intensity (Isshiki, 1965). Whether vocalists’ jaw movements alone affect observers’ perception of emotion is a topic for future research.

Expressive facial movements in all three facial features continued after sound production had ended. These movements consisted of sustained vertical lip corner contraction, inward brow furrowing (sadness-only), and opening of the jaw. Importantly, these extravocal movements occurred similarly in speech and song, supporting our hypothesis. The duration of these movements differed between the prevocal and postvocal epochs, with sustained emotional movements occurring throughout the postvocal epoch but occurring only briefly prior to the start of vocalization. We conducted a second experiment to determine the effect of facial movements on observers’ perception of emotion throughout the vocal timeline.

## EXPERIMENT 2

Experiment 2 tested the accuracy of observers’ perception of emotion from vocalists’ facial expressions that occurred prior to, during, and following speech and song vocalizations. Observers were asked to gauge the emotional intent based on silent video segments, which contained the vocalists’ facial expressions from only the timeline prior to, during, or after vocalization. In Experiment 1, systematic facial motion occurred prior to vocal onset and after vocalization ended, and movements that distinguished emotions were longer in duration in the postvocal epoch than the prevocal epoch. We hypothesized that emotions in speech and song would be identified on the basis of postvocalization facial movements with similar accuracy to judgements based on facial movements during vocalization, whereas judgements would be least accurate for facial movements occurring prior to vocalization.

### Method

#### Participants

Sixteen native English-speaking adults (8 male, mean age = 24.1 years, *SD* = 7.1), were recruited from the Montreal area. Participants were not chosen for their musical experience; they had received varied amounts of private music instruction (*M* = 7.0 years, *SD* = 5.9), singing experience (*M* = 3.19 years, *SD* = 6.0), and drama experience (*M* = 1.5 years, *SD* = 3.3). No participants from Experiment 2 participated in Experiment 1. Two highly trained female singers from Experiment 1 were recruited for stimulus creation. Singer 1 had 9 years of vocal experience, and Singer 2 had 10 years of experience. The participants received a nominal fee for their participation.

#### Stimulus and materials

The two singers were recorded while speaking or singing three neutral statements with the emotional intentions happy, neutral, and sad. The stimulus materials, design, and procedures for recording the two singers were identical to those used in Experiment 1, with the exception that no motion capture equipment was used, to record videos of facial expression without any markers, and that only three emotions were used (happy, neutral, sad). The singers were recorded with a JVC Everio GZ-HD6 camera and an AKG C 414 B-XLS cardioid microphone, placed 1.5 m in front of the vocalists at 44 kHz. The singers stood in front of a green-screen cloth, illuminated with three Cameron Quartz Imager Q-750 lights with white diffusion parabolic umbrellas. This setup provided natural-spectrum lighting, while eliminating facial shadows caused by overhead lighting.

**Figure 4:**
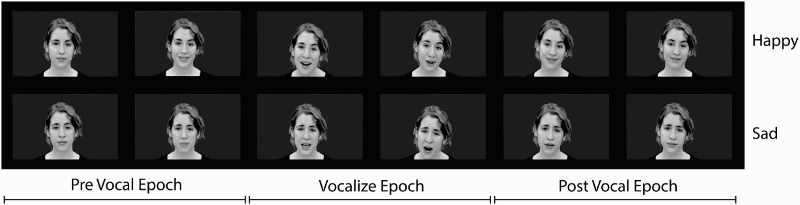
Still images from happy (top row) and sad (bottom row) silent movie stimuli used in Experiment 2, showing the three epochs of vocal communication. Boundaries between prevocal and vocalize epochs, and vocalize and postvocal epochs were determined by the onset and offset of vocal sound respectively.

The singers’ recordings were divided into three epochs: prevocal (1.90 s prior to vocal onset), vocalize (vocal onset to vocal offset, mean duration = 2.05 s; speech mean = 1.62 s, song mean = 2.48 s), and postvocal (1.90 s after vocal offset), as shown in Figure 4. A prevocal and postvocal epoch duration of 1.90 s was selected so that no speech vocalize-epoch stimuli (maximum duration = 1.9 s) were longer than any prevocal or postvocal speech epoch stimuli. Vocal epochs were marked using Praat (Boersma & Weenink, 2010), and recordings were edited using Adobe Premiere Elements. Video-only presentations (no audio) were presented to participants using E-Prime software.

#### Design, procedure, and analyses

The experimental design was a Channel (2 levels: speech or song) × Emotion (3 levels: happy, neutral, sad) × Epoch (3 levels: prevocal, vocalize, postvocal) × Statement (3) × Repetition (2) within-subjects design, with 108 trials per participant. Trials were blocked by channel, and order of channel was counterbalanced across participants, with emotion, epoch, statement, and repetition presented in a pseudorandom order within each block. On each trial, participants were asked to identify the emotional intent of the vocalist using a forced-choice categorical response measure (happy, neutral, and sad). Prior to each block, participants began with practice trials in which statements not used in the experimental trials were presented for that channel condition. Participation in the experiment took approximately 30 min.

Raw accuracy scores were converted to unbiased hit rates (Wagner, 1993). Unbiased hit rate corrects for possible response bias in categorical response tasks while allowing for multilevel designs (referred to as hit rate hereafter). As hit rates are proportion scores (0–1), data were arcsine square root transformed prior to statistical analysis (Wagner, 1993). For ease of readability, pretransformed hit rate means (0–1) are reported in both the body text and figures. The factors statement and repetition were collapsed prior to analysis. Hit rate scores were analysed with a repeated measures ANOVA. When Mauchly's sphericity test was significant, Greenhouse–Geisser's correction was applied. All effect sizes report partial eta-squared values. All statistical tests were conducted in Matlab 2013b and SPSS v20.0.0.

### Results

**Figure 5:**
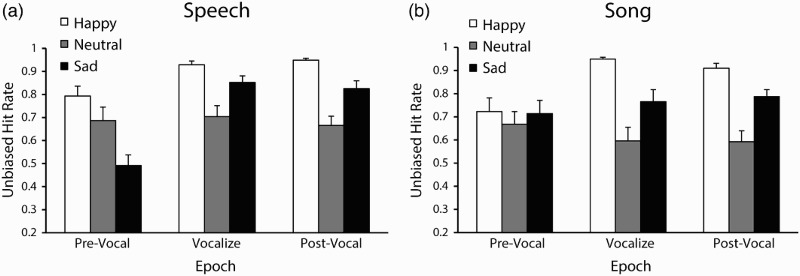
Mean unbiased hit rates by emotion and epoch in Experiment 2 for Speech and Song. Error bars denote the standard error of the means.

Participants’ mean unbiased hit rates are shown in Figure 5. A three-way ANOVA by channel (2), emotion (3), and epoch (3) was conducted on participants’ hit rate scores. No effect of channel was found, confirming that speech and song were identified with comparable recognition accuracy. A significant main effect of emotion was reported, *F*(2, 30) = 49.3, *p* < .001, 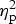 = .77. Post hoc comparisons (Tukey's honestly significant difference, HSD = .08, *α* = .05) confirmed that happy, *M* = .88, 95% confidence interval, CI [.83, .92] was identified significantly more accurately than sad, *M* = .74, 95% CI [.70, .81], and that both emotions were identified more accurately than neutral, *M* = .65, 95% CI [.57, .74]. A main effect of epoch was also reported, *F*(2, 30) = 40.96, *p* < .001, 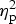 = .73. Post hoc comparisons (Tukey's HSD = .05, *α* = .05) confirmed that emotions in the prevocal epoch *M* = .68, 95% CI [.60, .76] were identified significantly less accurately than those during the vocalization, *M* = .80, 95% CI [.74, .86], and Postvocal epochs, *M* = .79, 95% CI [.74, .84], supporting our hypothesis that emotions for postvocalize movements would be identified at or near the accuracy for vocalize movements, and above those of prevocal movements.

A significant Channel × Epoch interaction, *F*(2, 30) = 5.12, *p* = .012, 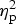 = .25, and significant Emotion × Epoch interaction, *F*(4, 60) = 12.19, *p* < .001, 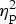 = .45, were reported. Post hoc comparisons (Tukey's HSD, *α* < .05) confirmed that speech–prevocal was identified less accurately than other speech epochs, and that song–prevocal was identified less accurately than the song–vocalize epoch. Happy and sad emotions were also identified less accurately in the prevocal epoch than during the vocalize and postvocal epochs, whereas accuracy for neutral remained unchanged across epochs. A significant Channel × Emotion × Epoch interaction was also observed, *F*(2, 30) = 5.44, *p* < .001, 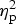 = .27, as shown in Figure 5. Post hoc comparisons (Tukey's HSD, *α* < .05) confirmed that speech–sad–prevocal was less accurate than all other channel–emotion–epoch conditions. These results indicate that observers’ recognition of emotion from prevocal movements was affected by the vocal channel and the emotion being expressed.

To determine whether recognition accuracy differences between epochs were mediated by differences in the amount of facial motion, a multiple linear regression was conducted to predict observers’ emotional accuracy scores from vocalists’ facial motion indicators recorded in Experiment 1. We selected vertical lip corner, horizontal brow, and Euclidean jaw motion as predictors, as these exhibited emotionally distinct movements throughout vocalization and postvocal epochs in Experiment 1. Mean displacement values were generated for each of the three epochs (prevocal, vocalize, postvocal). Viewers’ accuracy scores were regressed on the mean absolute displacements of the three motion trajectories (*n* = 108). The multiple regression analysis yielded a significant fit, *R* ^2^ = .20, *F* = 8.54, *p* < .001, with significant contributions of vertical lip corner motion, *β* = .25, *p* = .011, and horizontal brow motion, *β* = .31, *p* = .002. These results indicate that the extent of facial motion affected observers’ accuracy, with greater facial displacement of lip corners and eyebrows leading to higher rates of accuracy for identifying emotion.

The stimuli from the vocalize epoch varied in duration between speech and song conditions, and in comparison to pre- and postvocal stimulus durations. Although this difference did not affect emotional accuracy scores, it may have affected the speed with which observers made their emotional identification. To assess this relationship, a three-way ANOVA by channel (2), emotion (3), and epoch (3) was conducted on participants’ judgement response times (although subjects were instructed to respond after each stimulus ended, we interpreted shorter responses to indicate ease of judgements). No effect of channel was found, confirming that observers identified the vocalized emotion with comparable latency across speech and song. No effect of emotion was found. Interestingly, a main effect of epoch was reported, *F*(2, 30) = 7.6, *p* = .002, 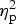 = .34. Pairwise comparisons confirmed that response times for the prevocal epoch, *M* = 1422.33 ms, 95% CI [1205.52, 1639.13], were significantly longer than those for both the vocalize epoch, *M* = 1234.5 ms, 95% CI [1068.88, 1400.11], and the postvocal epoch, *M* = 1232.35 ms, 95% CI [1071.49, 1393.22]. These results confirm that while observers were slower to identify prevocalization emotions, no differences were found in response time between speech and song.

### Discussion

Facial movements that occurred during and after vocalists’ sound production most accurately conveyed emotion to observers. As hypothesized, participants identified emotional expressions based on facial movements that occurred after vocal sound had ended with equivalent accuracy to facial movements during vocalization, while emotions based on movements prior to sound production were identified least accurately. These findings support the theory that emotional expression following vocalization may function to support the just-vocalized emotional message in speech and song.

In Experiment 1, vocalists’ facial movements following vocalization continued up to 2400 ms after vocal sound had ended, reflecting the gradual relaxation of facial muscles to a resting baseline. Given the duration over which relaxation occurred, postvocal expressions in Experiment 2 may have borne some similarity to static expressions of emotion. However, postvocal movements are qualitatively different from static images. Unlike static facial expressions, the speed of muscle relaxation in postvocal movements is likely to be important for correctly identifying emotion. For example, a rapidly falling smile may lead to a misinterpretation of the intended happy emotion. Thus, observers must strike a balance between recognizing the static representation of the emotion, and understanding the movements not as an emotional reaction but rather as a relaxation of an existing emotion.

Emotions were identified most accurately for happiness, followed by sadness, and then neutral emotional intent. These differences follow effects commonly reported in the literature for dynamic and static silent facial expressions, in which happiness is typically identified more accurately than sad expressions (Kohler et al., 2004; Scherer, 2003). Emotional expressions contained in facial movements during speech and song were identified with similar rates of recognition accuracy. Interactions between emotion and vocal channel were driven by reduced accuracy for sad prevocal movements in speech. Aside from this effect, these findings support the hypothesis that observers decoded emotion at similar rates of accuracy from expressive movements occurring after vocalization had ended. Observers’ recognition accuracy were also correlated with vocalists’ lip corners and eyebrow displacements reported in Experiment 1, but not jaw motion. These two facial features are commonly reported in the literature as primary indicators of happy and sad emotions in nonvocal facial expressions (Kohler et al., 2004).

The first two experiments have established that vocalists’ dynamic facial cues to emotion accurately convey emotion in speech and song. We conducted a third experiment to evaluate the relative contributions of visual and auditory expressions in vocal communication.

## EXPERIMENT 3

Experiment 3 examined observers’ perception of emotion from audio-only, video-only, and full audio–video recordings of speech and song. It is unknown how accurately facial expressions convey emotion relative to the voice during vocal communication. Previous research suggests that emotions are identified more accurately from visual information than auditory signals. We hypothesized that emotions would be identified least accurately for audio-only productions. We addressed this hypothesis by asking participants to identify the emotion from recordings of emotional speech and song in the three modality conditions audio-only, video-only, and full audio–video. To ensure comparisons of equal duration across modality conditions, all trials contained only the time region during which sound was vocalized; pre- and postvocal movements were not included as no sound is present during these epochs.

### Method

#### Participants

Sixteen native English-speaking adults (8 male, mean age = 22.8 years, *SD* = 3.5) were recruited from the Montreal area. Participants had received varied amounts of private music instruction (*M* = 3.1 years, *SD* = 4.6), singing experience (*M* = 1.9 years, *SD* = 3.1), and drama experience (*M* = .8 years, *SD* = 1.1). No participants from Experiment 3 had participated in Experiments 1 or 2. Participants received a nominal fee for their participation.

#### Stimulus and materials

Video recordings of the vocalize epoch recorded for Experiment 2 were used in Experiment 3. The recordings were exported to three modality conditions: audio-only, video-only, and full audio–video (AV; see online supplemental material). The vocalize epoch, defined as the onset of vocal sound to the offset of vocal sound, was chosen to keep stimulus length matched across all modality conditions. The duration of the vocalize epoch differed across channels and slightly across statements; speech mean duration = 1.7 s, *SD* = 0.25, song mean duration = 2.61 s, *SD* = 0.15. Recordings were edited using Adobe Premiere Elements, and stimuli were presented to participants using E-Prime software, over closed headphones (AKG K271).

#### Design, procedure, and analyses

The experimental design was a Channel (2 levels: speech or song) × Emotion (3 levels: happy, neutral, sad) × Modality (3 levels: audio-only, video-only, full-AV) × Statement (3) × Repetition (2) within-subjects design, with 108 trials per participant. Trials were blocked by channel and counterbalanced across participants, with emotion, modality, statement, and repetition presented in a pseudorandom order within each block. On each trial, participants were asked to identify the emotion of the performer using a forced-choice categorical response measure (happy, neutral, and sad). Prior to each block, participants began with practice trials in which statements not used in the experimental trials were presented for that channel condition. Participation in the experiment took approximately 30 minutes.

Raw accuracy scores were converted to unbiased hit rates (Wagner, 1993), as was done in Experiment 2. As hit rates are proportion scores (0–1), data were arcsine square root transformed prior to statistical analysis. For ease of readability, pretransformed hit rate means (0–1) are reported in the body text and figures. The factors statement and repetition were collapsed prior to analysis. Hit rate scores were analysed with a repeated measures ANOVA. When Mauchly's sphericity test was significant, Greenhouse–Geisser's correction was applied. All effect sizes report partial eta-squared values. All statistical tests were conducted in Matlab 2013b and SPSS v20.0.0.

### Results

**Figure 6:**
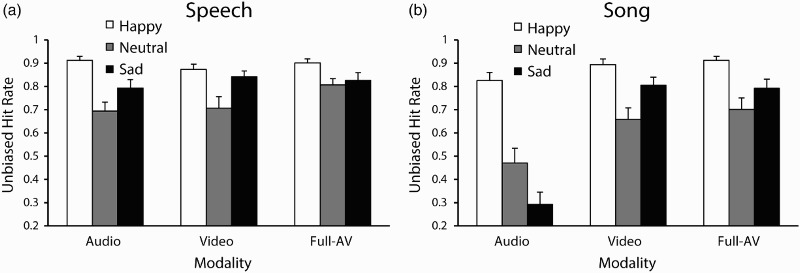
Mean unbiased hit rates by Modality and Emotion in Experiment 3 for (a) Speech and (b) Song. Error bars denote the standard error of the means.

Participants’ mean unbiased hit rates are shown in Figure 6. A three-way ANOVA by channel, emotion, and modality was conducted on participants’ hit rate scores. A significant main effect of channel was reported, *F*(1, 15) = 40.46, *p* < .001, 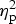 = .73, with speech, *M* = .82, 95% CI [.78, .86], identified significantly more accurately than song, *M* = .71, 95% CI [.66, .75]. A main effect of emotion was also reported, *F*(1.17, 16.85) = 31.15, *p* < .001, 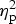 = .68. Post hoc comparisons (Tukey's HSD = .09, *α* = .05) confirmed that happy, *M* = .89, 95% CI [.85, .92], was identified significantly more accurately than sad, *M* = .73, 95% CI [.68, .77], and neutral, *M* = .67, 95% CI [.61, .74]. Importantly, a main effect of modality was reported, *F*(1.12, 16.85) = 25.99, *p* < .001, 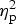 = .63. Post hoc comparisons (Tukey's HSD = .07, *α* = .05) confirmed that audio-only, *M* = .67, 95% CI [.61, .72], was identified significantly less accurately than video-only, *M* = .80, 95% CI [.75, .84], and full-AV, *M* = .82, 95% CI [.79, .87].

A significant Channel × Emotion interaction, *F*(2, 30) = 11.28, *p* < .001, 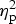 = .43, was reported. Post hoc comparisons (Tukey's HSD = .1, *α* = .05) confirmed that happiness was recognized with comparable accuracy in speech, *M* = .90, 95% CI [.86, .93], and song, *M* = .88, 95% CI [.83, .92], whereas neutral, *M*_speech_ = .74, 95% CI [.68, .80], *M*_song_ = .61, 95% CI [.53, .69], and sad, *M*_speech_ = .82, 95% CI [.76, .88], *M*_song_ = .63, 95% CI [.57, .69], were recognized more accurately in speech than in song. A significant Channel × Modality interaction was also reported, *F*(2, 30) = 27.09, *p* < .001, 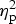 = .64. Post hoc comparisons (Tukey's HSD = .1, *α* = .05) confirmed that song–audio-only, *M* = .53, 95% CI [.45, .61], was significantly less accurate than speech–audio-only, *M* = .80, 95% CI [.75, .85], song–video-only, *M* = .79, 95% CI [.74, .84], and song–full-AV, *M* = .80, 95% CI [.75, .86]. No significant differences were found between the speech conditions. A significant Emotion × Modality interaction was also reported, *F*(4, 60) = 10.06, *p* < .001, 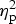 = .40, as was a significant Channel × Emotion × Modality interaction, *F*(4, 60) = 5.86, *p* < .001, 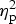 = .28, as illustrated in Figure 6. Post hoc comparisons (Tukey's HSD, *α* < .05) confirmed that song–neutral–audio-only and song–sad–audio-only were less accurate than all other channel–emotion–modality trials. For video-only and full-AV, no significant differences were reported between speech and song within each emotion. These results indicate that observers’ recognition of emotion from audio-only singing was affected by the emotion being expressed, and that in video-only and full-AV conditions, speech and song conveyed emotion at similar levels of accuracy. To determine whether observers’ accuracy scores for song–sad–audio-only, *M* = .29, 95% CI [.18, .41], differed from chance, a one-sample *t*-test with a chance estimate of .33 was conducted. Observers’ accuracy of emotional identification for song–sad–audio recordings, *M* = .29, 95% CI [.18, .41], was not significantly different from chance levels, *t*(95) = 0.49, *p* = .62, 95% CI [−.07, .12].

As in Experiment 2, stimuli varied in duration between speech and song. To assess whether this difference affected emotional accuracy scores, a three-way ANOVA by channel (2), emotion (3), and modality (3) was conducted on participants’ judgement response times (again, we interpreted faster responses to indicate ease of judgements). No effect of channel was found, confirming that observers identified the vocalized emotion with comparable latency across speech and song. No effect of emotion was found. A main effect of modality was reported, *F*(1.14, 17.06)  = 8.11, *p* = .009, 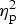 = .351. Post hoc comparisons (Tukey's HSD = 202.65, *α* = .05) confirmed that audio-only, *M* = 1434.61 ms, 95% CI [1213.55, 1655.67], were identified significantly slower than video-only, *M* = 1130.3 ms, 95% CI [987.72, 1272.88], and full-AV, *M* = 1170.54 ms, 95% CI [1029.05, 1312.04]. A significant Channel × Modality interaction was also reported, *F*(1.14, 17.06) = 8.11, *p* = .009, 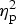 = .35. Post hoc comparisons (Tukey's HSD = 335.06, *α* = .05) confirmed that song–audio-only was identified significantly more slowly than all other conditions. These results corroborate hit rate findings, for which observers were both slower to respond and less accurate for singing audio-only presentations.

### Discussion

Video-only and audiovisual recordings of facial expressions during vocalizations conveyed emotion more accurately than the acoustic recordings alone, supporting the main hypothesis. The worst overall performance in recognition accuracy was for sad and neutral emotions in audio-only recordings of song. However, these conditions saw substantial improvements in recognition accuracy with the addition of visual information. While observers were not better than chance at identifying emotion from audio-only presentations of sad song, they were highly accurate at identifying vocalists’ intended emotion in full-AV and video-only presentations of sad song. Speech and song were identified at comparable levels of accuracy for video-only and audio-visual conditions. Emotion recognition accuracy for audio-only recordings of speech was higher than is typically reported (Scherer, 2003). This was perhaps due to a smaller range of emotion response options and reflects a ceiling effect.

Emotion recognition accuracy for audio-only presentations of neutral and sad emotions was significantly lower for song than for the equivalent speech presentations. This finding may not mean that the singing voice cannot accurately convey emotional information; instead, it may derive from influences of the predetermined musical composition on emotional expression. The structure of a musical composition, separate from its performance, is an important component of listeners’ perceived emotion (Gabrielsson & Lindström, 2001). The musical features pitch height, pitch variability, tempo, and mode strongly influence listeners’ perception of emotion in music (Hevner, 1935; Livingstone, Muhlberger, Brown, & Thompson, 2010; Thompson & Robitaille, 1992). Given the use of a fixed melody across emotions, singers’ range of manipulable acoustic features was reduced in comparison to speech, where pitch and duration are important acoustic cues to emotion in speech (Cowie et al., 2001; Scherer, 2003).

## GENERAL DISCUSSION

Three experiments provided evidence of broad commonalities in the dynamic facial cues to emotion in the production and perception of speech and song. Vocalists exhibited characteristic movements of the eyebrows and lip corners that transcended lexical and speech–song differences. These expressive movements corresponded to prototypical, silent expressions of emotion, with a raising of the lip corners and eyebrows for happiness, and a furrowing of the brow in sadness (Ekman & Friesen, 1978; Kohler et al., 2004). As hypothesized, vocalists’ jaw motion exhibited channel-dependent (speech/song) and emotion-dependent differences. To the authors’ knowledge, this is the first evidence that motion of the jaw has been shown to differentiate emotional facial expressions during vocal communication. These variations appeared to be driven by differences in the acoustic signal between speech and song, and across emotions, where vocalists’ jaw motion was highly correlated with their vocal intensity (McClean & Tasko, 2003; Tasko & McClean, 2004). These differences in jaw motion did not appear to affect emotional perception, as observers’ accuracy of emotional identification was positively correlated with vocalists’ lip corner and eyebrow displacement, but not with jaw displacement. Collectively, these results suggest that speech and song have broad commonalities in the dynamic facial cues to emotional expression, corroborating related findings in the overlap of acoustic cues to emotion in speech, song, and music (Ilie & Thompson, 2006; Juslin & Laukka, 2003; Scherer, 1995; Spencer, 1857). These findings also highlight that vocalists’ facial movements diverge in speech and song for movements that are tightly coupled to acoustic production.

Vocalists exhibited dynamic facial movements that extended beyond the time window of vocalization, with sustained vertical lip corner raising and opening of the jaw in happiness and sustained inward furrowing of the brow in sadness. These movements presented similarly in speech and song, supporting the hypothesis that extravocal facial movements are a general property of vocal communication (Livingstone et al., 2009). Extravocal movements prior to vocal onset began up to 500 ms before sound production, with motion trajectories moving away from a resting baseline, reflecting facial muscle contraction. In contrast, postvocal movements continued up to 2400 ms after vocal sound had ended, with motion trajectories returning to a resting baseline, reflecting facial muscle relaxation. These differences probably reflect the distinct roles of these movements; prevocal movements are the rapid facial muscle contractions that occur in the initial formation of expressions accompanying vocal sound, while postvocal movements are an intentionally slow relaxation of facial expressions to clarify the just-vocalized acoustic signal. Perceptual findings supported this hypothesis, where movements occurring after vocalization were identified with a high level of accuracy that was comparable to expressions occurring during vocal sound production, while prevocal expression were identified least accurately. Importantly, the perceptual results supported the motion findings of Experiment 1, where vocalists’ facial expressions were identified with comparable accuracy in speech and song, during vocalization and after vocal offset. These results provide further evidence that speech and song express emotion with similar patterns of facial movements that extend across the full timeline of vocalization.

Visual cues to emotional expression during singing performance conveyed emotion more accurately than the acoustic signal alone. Emotions in song were identified least accurately in the acoustic modality and with comparable accuracy in the video-only and audiovisual conditions. This finding suggests that observers’ identification of emotion from audiovisual presentations was driven primarily by information contained in the visual modality. Importantly, the addition of visual information significantly improved the recognition of emotion in song, achieving comparable accuracy to speech. Collectively, these results suggest that facial expressions can play an important role in supporting or clarifying the acoustic signal (Davidson, 1993; Elfenbein & Ambady, 2002; Vines et al., 2011). In speech, vocalists conveyed emotion with equivalent accuracy across all modality conditions. This may reflect a ceiling effect due to a small range of emotion response options. Overall, these results provide partial support for our hypothesis that facial expressions convey emotion more accurately than the voice in vocal communication.

The present study was designed to capture expressions of emotion that approximated those in a multiperson environment. To induce the mental and physiological correlates of emotion, experienced vocalists were asked to prepare themselves emotionally as they would for performing in front of others. The effect of the emotional induction procedure on vocalists’ productions was not assessed in the current study. Future work may compare such induction procedures with other emotional induction methods, for example by assessing responses in front of an audience or by comparison with a no-induction condition. The use of induction controls is gaining use amongst researchers who seek ecologically valid recordings of emotion in a laboratory setting (Bänziger, Mortillaro, & Scherer, 2012; Douglas-Cowie et al., 2007; Livingstone, Choi, & Russo, 2014).

## CONCLUSION

Speech and song have historically been regarded as overlapping and interchangeable forms of vocal communication. Studies have highlighted similarities in the acoustic cues to emotion in speech and song, overlooking parallels in the visual domain. This study highlighted that speech and song share broad similarities in the production and perception of facial movements tied to emotional expression across the timeline of communication, yet differed in movements coupled to sound production. These facial expressions were found to play an important supporting role, clarifying deficits in the acoustic modality. These findings extend our understanding of the entwined nature of speech and song to the visual domain, highlighting their use as overlapping and interchangeable forms of vocal expression.

### Supplemental Material

Supplemental material for this article is available via the “Supplemental” tab on the article's online page (http://dx.doi.org/10.1080/17470218.2014.971034).

## Footnotes

1 Some schools of classical performance train vocalists to inhibit facial motion. We screened participants prior to testing and excluded anyone who had received this form of training. We also screened vocalists who had been trained to express emotions with a particular facial expression.

2 The vocalize epoch contains 600 data samples (2.4 s at 250 Hz).
